# Machine learning algorithm predicts urethral stricture following transurethral prostate resection

**DOI:** 10.1007/s00345-024-05017-x

**Published:** 2024-05-15

**Authors:** Emre Altıntaş, Ali Şahin, Huseyn Babayev, Murat Gül, Ali Furkan Batur, Mehmet Kaynar, Özcan Kılıç, Serdar Göktaş

**Affiliations:** 1 Faculty of Medicine, Department of Urology, Selcuk University, Tıp Fakültesi Alaeddin Keykubat Yerleşkesi Selçuklu, Konya, 42131 Turkey; 2 Faculty of Medicine, Selcuk University, Konya, Turkey; 3 Faculty of Medicine, University of Zurich, Zurich, Switzerland

**Keywords:** Urethral stricture, Transurethral prostate resection, Machine learning,blood parameters

## Abstract

**Purpose:**

To predict the post transurethral prostate resection(TURP) urethral stricture probability by applying different machine learning algorithms using the data obtained from preoperative blood parameters.

**Methods:**

A retrospective analysis of data from patients who underwent bipolar-TURP encompassing patient characteristics, preoperative routine blood test outcomes, and post-surgery uroflowmetry were used to develop and educate machine learning models. Various metrics, such as F1 score, model accuracy, negative predictive value, positive predictive value, sensitivity, specificity, Youden Index, ROC AUC value, and confidence interval for each model, were used to assess the predictive performance of machine learning models for urethral stricture development.

**Results:**

A total of 109 patients’ data (55 patients without urethral stricture and 54 patients with urethral stricture) were included in the study after implementing strict inclusion and exclusion criteria. The preoperative Platelet Distribution Width, Mean Platelet Volume, Plateletcrit, Activated Partial Thromboplastin Time, and Prothrombin Time values were statistically meaningful between the two cohorts. After applying the data to the machine learning systems, the accuracy prediction scores for the diverse algorithms were as follows: decision trees (0.82), logistic regression (0.82), random forests (0.91), support vector machines (0.86), K-nearest neighbors (0.82), and naïve Bayes (0.77).

**Conclusion:**

Our machine learning models’ accuracy in predicting the post-TURP urethral stricture probability has demonstrated significant success. Exploring prospective studies that integrate supplementary variables has the potential to enhance the precision and accuracy of machine learning models, consequently progressing their ability to predict post-TURP urethral stricture risk.

## Introduction

Benign Prostatic Hyperplasia (BPH) presents a prevalent health concern impacting the overall well-being of men, typically manifesting in their 40 s and becoming more pronounced with age [1]. Severe BPH is notably prevalent in approximately 40% of men in their 60 s, and this proportion escalates to 50% as they advance in years [2].

Medical treatments are primarily used in the treatment of BPH. In cases where medical treatment is inadequate, surgical treatments such as resection or enucleation are planned [3]. Although different surgical methods, such as laser resection with Thulium, transurethral incision, transvesical open prostatectomy, and laser enucleation with holmium, are available, currently, the gold standard and the most frequently employed surgical method for BPH is transurethral resection of the prostate (TUR-P) [4]. Although TUR-P is recognized as an efficacious therapeutic approach, it is not exempt from prospective complications. Adverse outcomes after the procedure, such as incontinence, urethral stricture, and bladder neck stenosis, have the potential to significantly impact the patient's overall quality of life [5].

Urethral stricture may manifest in up to 10% of cases TUR-P, necessitating repeated surgical intervention [5–7]. While the precise mechanisms leading to the formation of urethral stricture remain uncertain, it is proposed that fibrosis emerges as a consequence of inflammatory processes occurring within the urethral epithelium and subepithelial tissues, ultimately resulting in stricture formation [8]. The occurrence of urethral stricture after transurethral TUR-P has been documented to be correlated with various risk factors. These include, but are not limited to, factors such as age, duration of the surgical procedure, prostate volume, the expertise of the surgeon, and the presence of infection [9]. Given the pivotal role of inflammation in the pathogenesis of urethral stricture, recent studies have concentrated their efforts on investigating this aspect. These studies aim to predict the risk of urethral stricture by quantifying inflammation levels on an individual basis [10–13].

Despite all these studies, there is currently no established marker or method capable of predicting the inflammation related to the development of urethral stricture after TUR-P. In recent years, there has been a growing prevalence of employing artificial intelligence and machine learning algorithms in the realm of healthcare. Machine learning is increasingly harnessed for the interpretation of patient images and the prediction of potential pathologies through the analysis of patient data [14, 15]. In light of these advancements, we aimed to predict the development of postoperative urethral stricture by evaluating the data obtained from preoperative blood parameters with different machine learning algorithms in patients undergoing TUR-P for BPH.

## Methods

Approval for this study was obtained from Selçuk University Faculty of Medicine Ethics Committee (Decision no: 2023/16).

### Study population

We retrospectively analyzed data from patients who underwent bipolar TUR-P using the GyrusPlasmaKineticTM system (Gyrus ACMI, USA) from January 2015 to January 2022, with ethical approval from the local ethics committee. To minimize bias, cases performed by two urologists with at least five years of TUR-P experience were included. Data included patient characteristics, preoperative blood test results, and post-surgery uroflowmetry outcomes. Parameters such as age, prostate-specific antigen (PSA) levels, prostate volume, and perioperative operation times were recorded. TUR-P utilized a standard continuous irrigating resectoscope with a 26 French outer sheath, and plasmakinetics settings were 200 W for shearing and 120 W for coagulation. A tri-way 20 F Foley catheter remained for 2–4 days post-surgery. Discharged patients underwent uroflowmetry at 1, 3, and 12 months post-discharge.Endoscopy with flexible cystoscopy was performed under local anesthesia in patients in whom stenosis pattern was detected on uroflowmetry. Patients were not classified according to urethral stricture localization. All urethral strictures were confirmed by cystoscopy. Patients were grouped based on stricture presence. Exclusion criteria applied to those with urethral issues, bladder neck strictures, cancer treatments, hematologic disorders, infections during surgery, strictures at prior resection sites, and those receiving blood transfusions. In addition, patients with incomplete data after TUR-P and patients who underwent urological interventional procedures in another center after the operation were excluded from the study.

### Data collection

We gathered perioperative hematological parameters, PSA, alanine aminotransferase (ALT),aspartate aminotransferase (AST),activated partial thromboplastin clotting time, prothrombin time, international normalized ratio (INR), and age at TURP from patient records. Calculated indices included:

- De Ritis Ratio(DRR) = AST/ALT
- NLR = Neutrophil Count/Lymphocyte Count
- Mentzer Index(MI) = MCV/RBC
- PLR = Platelet Count/(Lymphocyte Count*1000)
- Lymphocyte/Monocyte Ratio (LMR) = Lymphocyte Count/Monocyte Count
- Systemic Inflammatory Response Index (SIRI) = (Neutrophil Count*Monocyte Count)/Lymphocyte Count
- Systemic Immune-Inflammatory Index (SII) = (Neutrophil Count*Platelet Count)/(Lymphocyte Count *1000)

### Machine learning

To predict urethral stricture development post-TUR-P for BPH, we used decision trees, logistic regression, random forests, support vector machine (SVM), k-nearest neighbors (k-NN), and naive Bayes algorithms. Data were split into 80% training and 20% test subsets for each model. Predictive performance was evaluated using F1 score, accuracy, negative predictive value, positive predictive value, sensitivity, specificity, Youden Index, ROC AUC value, and confidence interval. Python 3.11.5 with libraries, pandas 2.1.1, numpy 1.26.0, matplotlib 3.8.0, seaborn 0.12.0, and scikit-learn 1.3.1 were used for model implementation.

### Data visualization

To assess calibration and performance, we utilized ROC curves and confusion matrices for machine learning models. A heatmap, generated with Python libraries seaborn 0.12.0, numpy 1.26.0, and matplotlib 3.8.0 was employed to compare the effectiveness of all models.

### Statistical analysis

Descriptive statistical analyses for groups with and without urethral strictures were performed using Python libraries pandas 2.1.1 and scipy 1.11.2, calculating mean ± standard deviation values. The homogeneity of the data distribution was then evaluated using the Kolmogorov–Smirnov test. Statistical analyses were performed using t-test for the data that fit the normal distribution, while Mann–Whitney U-Test was used for the data that did not fit the normal distribution. p values of < 0.05 were deemed statistically significant.

## Results

### Patient characteristics

In this study, 615 patients were retrospectively analyzed and a total of 109 patients were included. This cohort was subsequently stratified into two distinct categories: a control group, which encompassed 55 patients who did not exhibit any signs of urethral stricture, and a study group, comprising 54 patients who received a confirmed diagnosis of urethral stricture.

Comprehensive data encompassing surgical age, hematological parameters, ALT, AST, PSA, coagulation parameters, as well as indices such as the NLR, PLR, DRR, Mentzer Index, LMR, SIRI, and SII are meticulously documented and presented in Table 1. Statistical analysis revealed no discernible statistical disparities between these two cohorts, except for preoperative Platelet Distribution Width (PDW), Mean Platelet Volume (MPV), Plateletcrit (PCT), Activated Partial Thromboplastin Time (APTT), and Prothrombin Time (PT) values. Furthermore, the median follow-up period after TURP and the median duration between the TURP procedure and the emergence of recurrence were computed at 12 months (with a range of 10 to 64 months) and 6.4 months, respectively.

**Table 1:** The clinical and laboratory results of the urethral stricture negative and urethral stricture positive

	US− (*n* = 55)	US + (*n* = 54)	*p* value
Age	69.38 ± 8.90	68.06 ± 7.81	0.41
NLR	2.79 ± 1.54	2.91 ± 1.97	0.72
PLR	132.82 ± 50.11	128.40 ± 48.47	0.64
DRR	1.21 ± 0.50	1.06 ± 0.46	0.10
Mentzer Index	18.15 ± 3.12	17.45 ± 2.46	0.19
LMR	3.38 ± 1.28	3.71 ± 1.51	0.23
SIRI	1.92 ± 1.42	1.59 ± 1.09	0.17
SII	708.42 ± 442.11	642.34 ± 390.01	0.41
WBC	8.19 ± 2.50	7.63 ± 2.06	0.21
HGB	14.37 ± 1.83	14.58 ± 1.28	0.47
HCT	43.07 ± 4.81	43.33 ± 3.91	0.75
PLT	251.75 ± 70.54	228.44 ± 56.72	0.06
RBC	4.93 ± 0.63	5.02 ± 0.50	0.40
MCV	87.69 ± 5.79	86.53 ± 5.47	0.28
MCH	29.23 ± 2.46	29.13 ± 2.22	0.81
MCHC	33.33 ± 1.52	33.66 ± 0.97	0.17
PDW	11.54 ± 2.41	16.56 ± 2.22	> 0.001*
MPV	9.85 ± 0.94	8.27 ± 1.18	> 0.005*
PCT	0.25 ± 0.07	0.19 ± 0.05	> 0.001*
NE#	5.19 ± 2.08	4.83 ± 1.74	0.33
LY#	2.08 ± 0.74	1.98 ± 0.74	0.48
MO#	0.66 ± 0.23	0.58 ± 0.27	0.09
EO#	0.20 ± 0.16	0.18 ± 0.15	0.61
BA#	0.06 ± 0.07	0.04 ± 0.04	0.26
NE%	61.98 ± 10.01	62.60 ± 10.83	0.75
LY%	26.61 ± 8.82	26.73 ± 8.88	0.94
MO%	8.17 ± 2.87	7.66 ± 2.61	0.33
EO%	2.41 ± 1.95	2.34 ± 1.56	0.84
BA%	0.63 ± 0.56	0.67 ± 0.41	0.66
PSA	8.04 ± 9.26	5.21 ± 8.05	0.09
ALT	17.60 ± 13.16	21.30 ± 10.70	0.11
AST	18.53 ± 9.14	19.65 ± 5.70	0.44
APTT	27.53 ± 2.60	26.09 ± 2.81	> 0.006*
PT INR	1.01 ± 0.07	1.03 ± 0.08	0.3
PT	9.12 ± 0.89	11.11 ± 0.92	> 0.001*

*US* Urethral Stricture, *NLR* Neutrophil-to-Lymphocyte Ratio, *PLR* Platelet-to-Lymphocyte Ratio, *DRR* De Ritis Ratio, *LMR* Lymphocyte-to-Monocyte Ratio, *SIRI* Systemic Immune-Inflammatory Index, *SII* Systemic Immune-Inflammation Index, *WBC* White Blood Cell, *HGB* Hemoglobin, *HCT* Hematocrit, *PLT* Platelet, *RBC* Red Blood Cell, *MCV* Mean Corpuscular Volume, *MCH* Mean Corpuscular Hemoglobin, *MCHC* Mean Corpuscular Hemoglobin Concentration, *PDW* Platelet Distribution Width, *MPV* Mean Platelet Volume, *PCT* Plateletcrit, *NE#* Neutrophil Count, *LY#* Lymphocyte Count, *MO#* Monocyte Count, *EO#* Eosinophil Count, *BA#* Basophil Count, *NE%* Neutrophil Percentage, *LY%* Lymphocyte Percentage, *MO%* Monocyte Percentage, *EO%* Eosinophil Percentage, *BA%* Basophil Percentage, *PSA* Prostate-Specific Antigen, *ALT* Alanine Aminotransferase, *AST* Aspartate Aminotransferase, *APTT* Activated Partial Thromboplastin Time, *PT INR* Prothrombin Time International Normalized Ratio, *PT*: Prothrombin Time *p < 0.05

There was no statistically significant difference between the two groups in terms of age (US−  = 69.38 ± 8.90 vs. US +  = 68.06 ± 7.81, p = 0,41), preoperative prostate volume (US- prostat volume = 72.75 ± 25.09 cc vs. US + prostate volume = 69,28 ± 21.71, p = 0.227) and operative time. (US−  = 66.6 ± 18.15 min vs. US +  = 62.06 ± 11.28 min, p = 0.12). An analysis of the hematological parameters revealed significant disparities in PDW (16.56 ± 2.22 vs. 11.54 ± 2.41; p < 0.001) and PT (11.11 ± 0.92 vs. 9.12 ± 0.89; p < 0.001) levels in patients who developed urethral stricture compared to those who did not exhibit this condition (Table 1). In stark contrast, patients devoid of urethral stricture showcased noteworthy elevations in MPV (9.85 ± 0.94 vs. 8.27 ± 1.18; p < 0.001), PCT (0.25 ± 0.07 vs. 0.19 ± 0.05; p < 0.001), and APTT (27.53 ± 2.60 vs. 26.09 ± 2.81; p = 0.006) levels, when scrutinized against their counterparts who eventually developed urethral stricture (Table 1). On the other hand, there was no statistically significant difference between the two groups in terms of age, preoperative prostate volumes and operation times.

### Machine-learning models

**Fig. 1:**
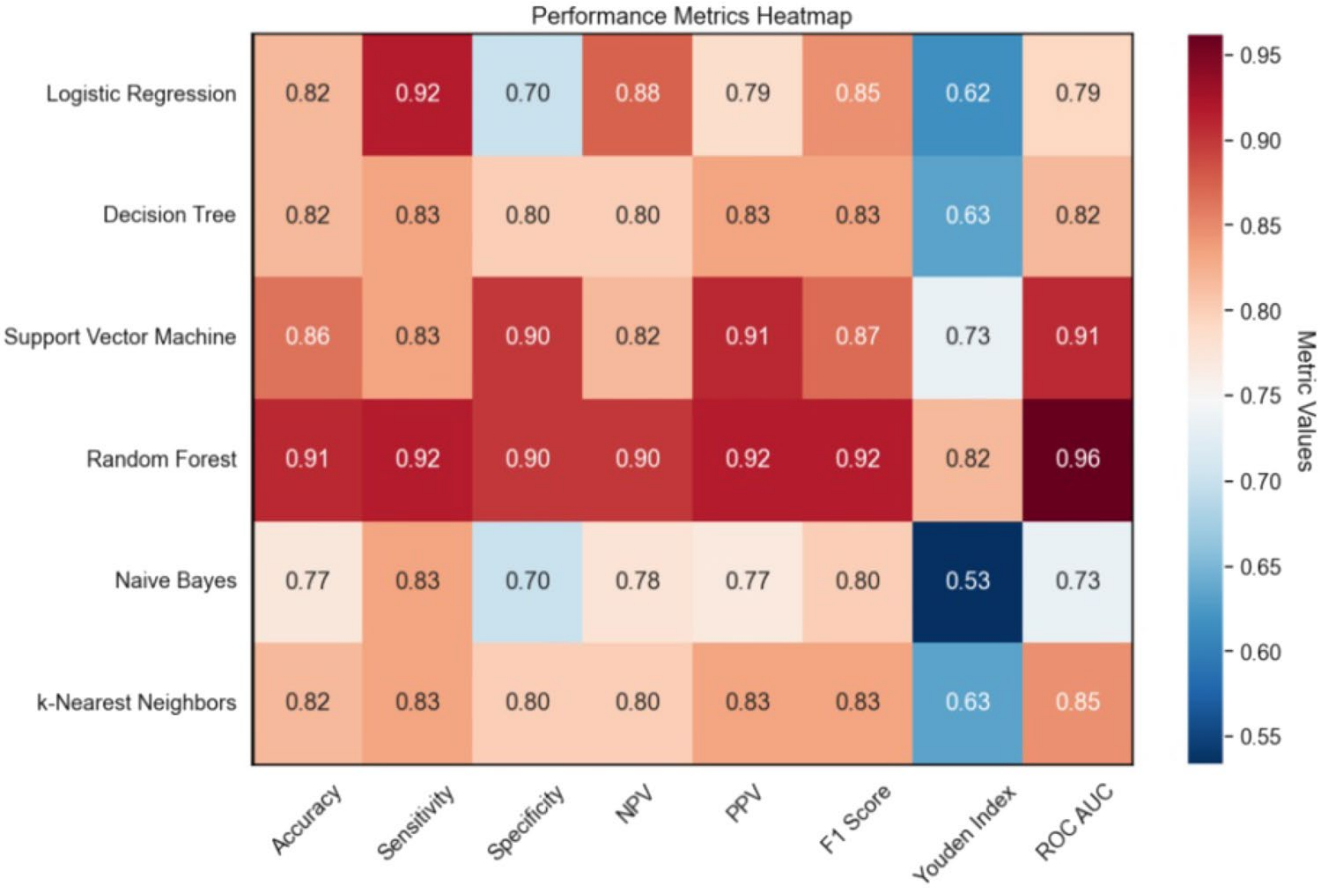
Machine learning model performance metrics heatmap

**Fig. 2:**
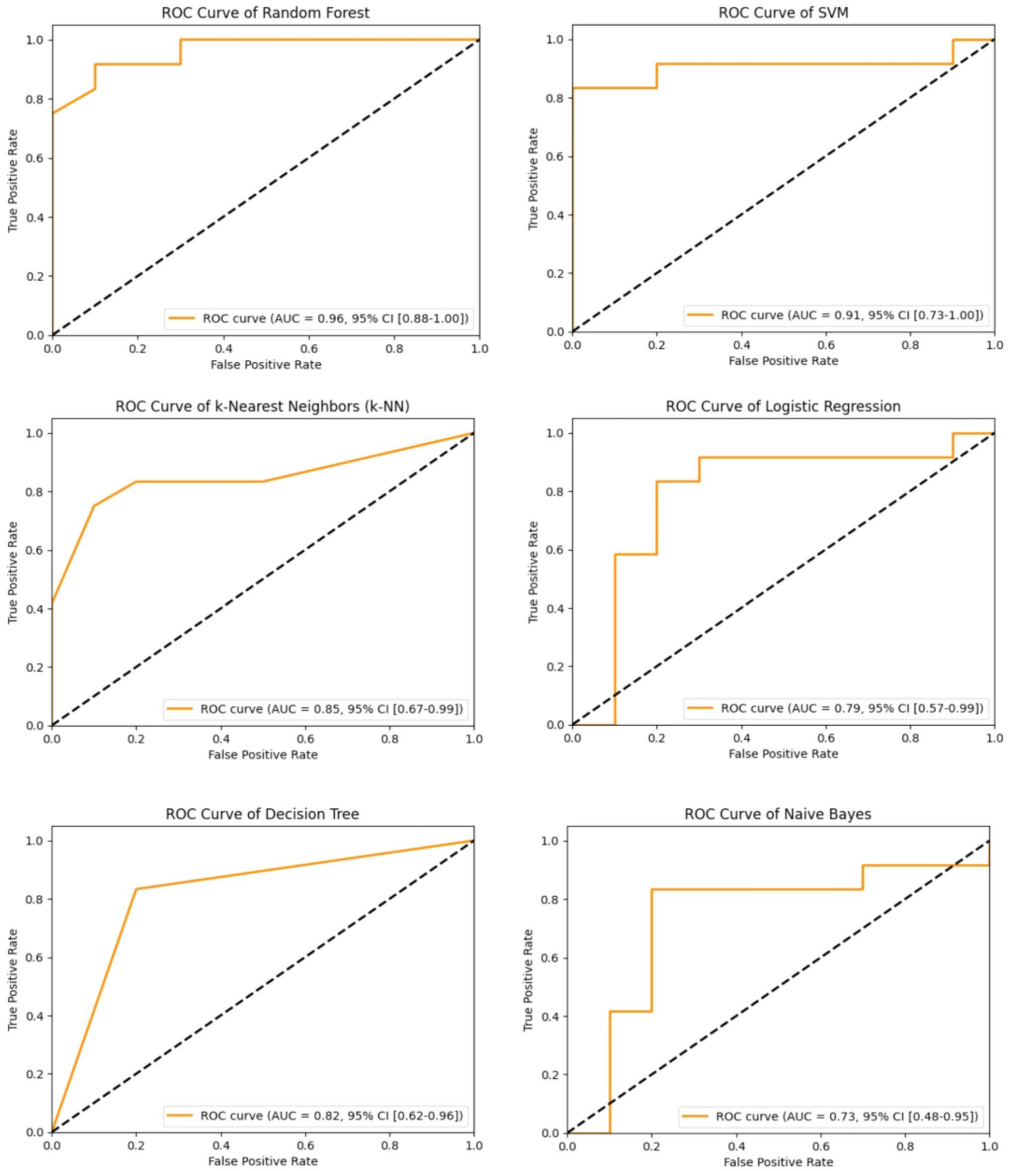
ROC curves of machine learning models

A comprehensive predictive modeling effort used various machine learning algorithms, including decision trees, logistic regression, random forests, SVM, k-NN, and naive Bayes. These models aimed to predict the likelihood of urethral stricture after TUR-P using a robust dataset with 35 patient-specific variables. The predictive performance of each model was evaluated, and results are summarized in Fig. 1. Accuracy scores for the algorithms were as follows: decision trees (0.82), logistic regression (0.82), random forests (0.91), SVM (0.86), k-NN (0.82), and naive Bayes (0.77). Corresponding AUC values from ROC curves were:decision trees (0.82), logistic regression (0.79), random forests (0.96), SVM (0.91), k-NN (0.85), and naive Bayes (0.73), emphasizing the discriminative potential of these models (Fig. 2).

**Fig. 3:**
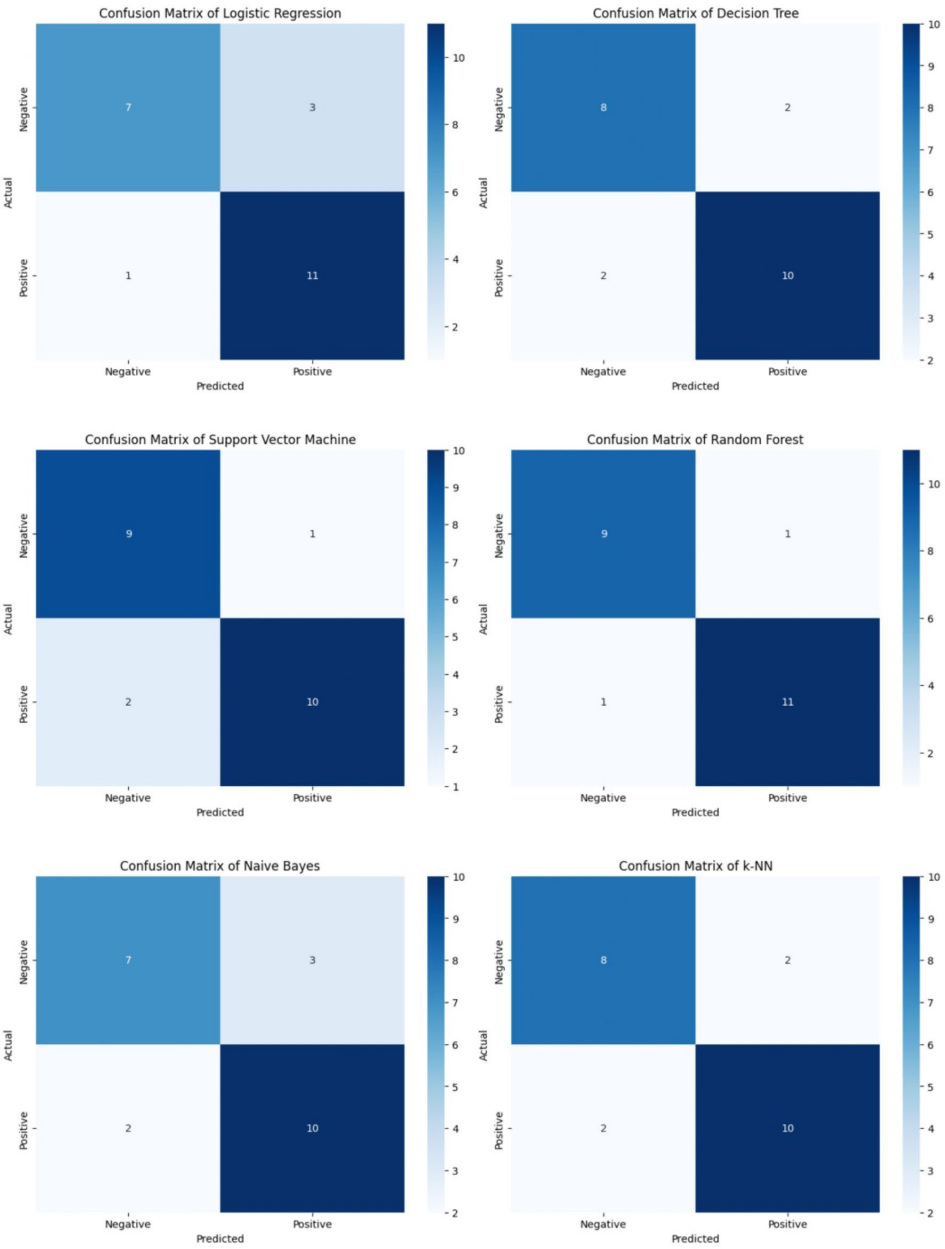
Confusion matrices of machine learning algorithms

To thoroughly evaluate machine learning models predicting urethral stricture development, confusion matrices (Fig. 3) were included. These matrices offer a detailed breakdown of true positives, true negatives, false positives, and false negatives. This provides a nuanced view of model accuracy and discriminatory capability in identifying urethral stricture cases.

## Discussion

Urethral stricture incidence post-TUR-P ranges from 2.2% to 9.8%, posing significant challenges [5]. Higher risk is noted in those with prior endoscopic urologic surgery. The propensity for urethral stricture after TUR-P is not fully understood. While prior studies explored blood parameters as predictors, our study is the first to integrate these parameters with machine learning for a comprehensive analysis.

Fibroblasts primarily contribute to urethral stricture development, with scarring arising from heightened inflammation due to urine extravasation and minimal bleeding [10]. In urethroplasty specimens, 44% showed chronic inflammation, suggesting inflammation as a key factor [10]. Studies have explored parameters like PLR, NLR, and SII as indicators of inflammation.

Plasma NLR and PLR, nonspecific markers of systemic inflammation, were studied for their role in urethral stricture development post-TUR-P. A 2016 study on PLR's predictive efficacy for anterior urethral strictures reported 84% sensitivity and 64% specificity, focusing exclusively on anterior strictures from TUR-P [12]. In our inclusive study covering all urethral strictures post-TUR-P, PLR showed no significant difference between groups, and NLR did not exhibit a significant relationship. Another 2018 study found increased NLR values in patients with recurrent urethral stricture post internal urethrotomy, compared to those without stricture [13].

A recent investigation found higher SII levels in recurrent urethral stricture cases, with a reported sensitivity of 59.62% and specificity of 70.41% [16]. However, our study observed no statistically significant differences in PLR, NLR, SII, MI, DRR, and SIRI values between the two groups. Notably, MPV and PCT were significantly higher in the group without urethral stricture (MPV: 9.85 ± 0.94 fL vs. 8.27 ± 1.18 fL, PCT: 0.25 ± 0.07 vs. 0.19 ± 0.05), while PDW values were notably higher in the urethral stricture group (PDW: 11.54 ± 2.41 fL vs. 16.56 ± 2.22 fL). These platelet-related parameter disparities between groups may hold significant predictive value for urethral stricture occurrence post-TUR-P.

Six machine learning algorithms were employed to predict urethral stricture development post-TUR-P. Unlike conventional statistical methods, machine learning algorithms excel in predicting and estimating medical conditions due to their ability to handle heterogeneous and interrelated medical data. Our study utilized preoperative laboratory data from patients with urethral stricture after TUR-P to construct a predictive model. Machine learning is widely applied in modern medicine, assisting in differential diagnoses, cancer staging, treatment response determination, and supporting healthcare professionals in various scenarios. In urology, these algorithms have been used to predict postoperative mortality after radical cystectomy for bladder cancer [17], evaluate surgical performance in robot-assisted radical prostatectomy [18], and identify biochemical recurrence post robot-assisted radical prostatectomy [19]. A 2022 study using machine learning on retrograde urethrography images accurately detected urethral stricture in 88.5% of cases. Another study successfully employed machine learning on endoscopic images for urethral and ureteral strictures, achieving a sensitivity of 0.96 [14, 15].

We utilized logistic regression, SVM, random forest, decision tree, k-NN, and naive bayes algorithms in our study. To assess model performance, we calculated various metrics, including ROC AUC values, negative predictive value, positive predictive value, Youden Index, sensitivity, specificity, F1 score, and accuracy. Random Forest demonstrated the highest accuracy (0.91), while Naive Bayes had the lowest (0.77). However, relying solely on accuracy may be misleading. The Youden Index, considering both sensitivity and specificity, is more appropriate. The Random Forest model had the highest Youden Index (0.82), while Naive Bayes had the lowest (0.53). Both models successfully discriminate, as their Youden Index values exceed 0.5. In terms of ROC AUC values, Random Forest achieved the highest (0.96), whereas Naive Bayes had the lowest (0.73).

Despite the retrospective nature of our study and its limited sample size, it holds potential significance in identifying patients at risk of developing urethral stricture during the preoperative phase prior to Transurethral Resection of the Prostate (TUR-P). By leveraging our findings, clinicians can proactively inform patients about the likelihood of urethral stricture development, enabling the formulation of preemptive treatment strategies aimed at averting such complications. Furthermore, our study stands out in the realm of medical research by pioneering the development of a machine learning model based on blood parameters rather than conventional focus on image processing. This innovative approach underscores the versatility and potential applicability of machine learning techniques beyond traditional domains, promising broader utility in clinical practice. We are optimistic that further refinement and validation of such models could pave the way for their integration into routine clinical workflows, thereby enhancing patient care and outcomes in the future.

The study has limitations, being single-center, retrospective, and conducted in a tertiary university hospital. Due to its retrospective design, inflammation markers couldn't be assessed, and immunohistochemical data from pathology samples are lacking. However, our focus was to enhance the understanding of the urethral stricture-inflammation relationship by including diverse indices and parameters like DRR, NLR, MI, PLR, LMR, SIRI, and SII.

## Conclusion

The utilization of machine learning models holds promise in the prediction of urethral stricture, a notable complication subsequent to TUR-P. We posit that the potential utility of such models is particularly pronounced in the context of risk stratification for individuals predisposed to developing urethral stricture following TUR-P. Future studies with larger cohorts and added parameters could enhance specificity and sensitivity, further advancing predictive capabilities for urethral stricture after TUR-P.
